# Perfluorinated Plastic Optical Fiber Tapers for Evanescent Wave Sensing

**DOI:** 10.3390/s91210423

**Published:** 2009-12-22

**Authors:** Roberto Gravina, Genni Testa, Romeo Bernini

**Affiliations:** Institute for Electromagnetic Sensing of the Environment (IREA), National Research Council (CNR), Via Diocleziano 328, 80124 Napoli, Italy; E-Mails: gravina.r@irea.cnr.it (R.G.); testa.g@irea.cnr.it (G.T.)

**Keywords:** plastic optical fiber, fiber taper, evanescent sensor

## Abstract

In this work we describe the fabrication and the characterization of perfluorinated plastic-cladded optical fiber tapers. The heat-and-pull procedure has been used to fabricate symmetric tapers. Devices with different taper ratio have been produced and the repeatability of the process has been verified. The very low refractive indexes of the core-cladding perfluorinated polymers (*n* = 1.35−1.34) permit a strong enhancement of the evanescent wave power fraction in aqueous environments (*n* = 1.33), making them very attractive for evanescent wave sensing. The tapers have been characterized carrying out evanescent field absorbance measurements with different concentrations of methylene blue in water and fluorescence collection measurements in an aqueous solution containing Cy5 dye. A good sensitivity, tightly related to the low refractive index of the core-cladding materials and the geometrical profile, has been shown.

## 1. Introduction

Fiber optic tapers are important devices that can act as sensors and couplers. The main feature of fiber tapers is the fact that they can strongly enhance the power fraction in the cladding in the form of evanescent wave increasing the sensitivity to environmental changes. Cladded and uncladded tapered optical glass fibers have been studied to obtain high sensitivity devices such as chemical sensors [1,2]. In particular, uncladded glass fiber tapers have been used for evanescent absorption measurements and fluorescence excitation/collection due to their strong evanescent field. However, after the removal of the cladding and the tapering of the glass fiber, the device is very fragile and requires careful control. Moreover, the tapering of core-exposed multimode fibers causes much of the guided light to be lost. Recently, to overcome these problems, the use of cladded glass fiber tapers has been proposed and demonstrated [2,3]. Despite the fact that the taper core is not in direct contact with the external medium, this fiber taper can be used for sensing applications. In fact, some of the guided modes are no longer confined in the core region, but can still be guided by the fiber in the cladding region. Therefore, in the taper region, there is an evanescent wave in the external medium, related to the cladding modes.

As an alternative to traditional glass, fiber plastic optical fibers (POFs) have attracted increasing interest in the last few years because of their interesting physical and mechanical features. POF will not break with strains of over 50%, whereas the silica-based fibres are fragile and will break under a strain of only 5%. The simplicity of use, related to simple end preparation and absence of expensive termination tooling, make POFs an excellent replacement for traditional glass fibers. Moreover, they show good tensile strength, load resistance, higher numerical aperture and lower bend radius limits than standard fibers.

However, it must be underlined that whereas the fabrication of fiber tapers in silica glass is a well-established process, both for sensing and communications applications, only few examples of plastic optical fiber (POF) tapers have been reported [4-6]. Typically POF tapers have been fabricated using chemical etching [4], in fact, due to low ductility and tendency for uneven melting, POF materials have previously been considered unsuitable for application of the heat-and-pull technique [4], but recently two examples of fabrication of POF tapers based on PMMA fibers using a heat-and-pull process have been reported [5,6]. However, until now, a detailed analysis of the fabrication and characterization of plastic optical fiber tapers fabricated by heat-and-pull technique has not been presented.

In this work we report for the first time an extensive fabrication and characterization of cladded perfluorinated graded index plastic optical fibers (GI-POFs) tapers produced by the heat-and-pull technique. The very low refractive indexes of the core-cladding perfluorinated polymers [7] (*n* = 1.35−1.34) allow a strong enhancement of the power fraction in the evanescent wave in aqueous environments (*n* = 1.33), making them very attractive in comparison with glass fiber tapers for sensing applications like biosensing or environmental monitoring [8-10]. In particular, we demonstrate that these devices can be used both for evanescent wave absorption and for evanescent wave fluorescence collection. Several devices with different taper ratios have been produced by the heat-and-pull technique. The repeatability of the process in terms of taper ratio and shape has been verified with good results. Finally, the sensing properties of the tapers have been characterized by carrying out evanescent wave absorption measurements in an aqueous solution containing methylene blue in a concentration range of 5 × 10^−8^ to 1 × 10^−6^ M and fluorescence measurements in an aqueous solution containing Cy5 dye in a concentration range of 3.14 × 10^−7^ M to 6.76 × 10^−6^ M.

## 2. Sensing Principle

**Figure 1.**
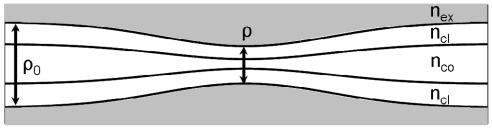
Schematic structure of a cladded fiber taper.

When a cladded optical fiber is tapered both the cladding and the core diameters are reduced in size. The schematic structure of a cladded taper is shown in Figure 1, where *n_co_, n_cl_* and *n_ex_* are the refractive indices of the fiber core, cladding and external medium, respectively. *ρ*_0_ and *ρ* are the diameters of the uniform fiber and the taper waist, respectively. The taper ratio is defined as *R* = *ρ/ρ*_0_ and L is the length of the taper. Despite to the fact that taper core is not in direct contact with the external medium, cladded fiber tapers can be used for sensing applications [2,3]. In fact, in the down taper region, some of the high-order guided modes are no longer confined in the core region but can still be guided by the fiber in the cladding region. Therefore, in the taper region, there is an evanescent wave related to cladding modes bounded by the cladding-external medium interface [2,3] that can be used for probing the absorption properties of the surrounding medium. At the end of the taper, some of the cladding modes are coupled back into guided core modes by the up-taper, hence the transmitted intensity can be detected at the proximal end of the fiber.

The penetration depth of these cladding modes is approximately given by:

(1) $$d_{p}=\frac{\lambda }{2\pi \sqrt{n_{\mathrm{cl}}^{2}\sin^{2}\theta -n_{\mathrm{ex}}^{2}}}$$

where λ is the wavelength of the light source, *θ* is the angle of incidence of the light at the cladding-external medium interface, *n_cl_* and *n_ex_* are the refractive indices of the cladding and external medium, respectively. From Equation (2) it is clear that the very low refractive index of the cladding perfluorinated polymer (1.34) permits a strong enhancement of the power fraction in the evanescent wave in aqueous environments (*n* = 1.33), in comparison with glass fibers (*n* ≈ 1.46).

It is very difficult to perform an experimental comparative study between fiber tapers fabricated with different materials because their optical (step index, graded index, input lunch angle,…) and geometrical (core/cladding diameters, length,…) parametersy also differ, so in order to permit a more accurate comparison between perfluorinated POF fibers and PMMA and glass fiber the theoretical absorbance has been calculated with the model developed by Gou and Albin. For the calculation, the authors used a ray-optic approach and considered a step-index multimode fiber [3].

**Figure 2.**
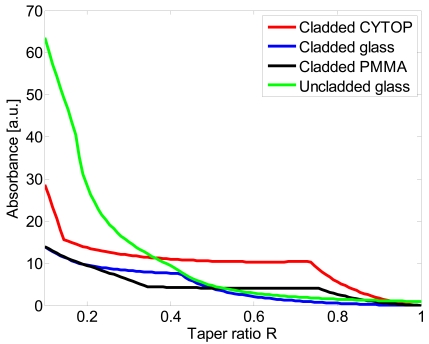
Absorbance of Cladded CYTOP, Cladded glass, Cladded PMMA and uncladded glass fiber taper.

The following data for the refractive indexes of core and cladding are used for the numerical analysis: *n_co_* = 1.469, *n_cl_* = 1.445 for glass fiber, *n_co_* = 1.492, *n_cl_* = 1.402 for PMMA fiber and *n_co_* = 1.356, *n_cl_* = 1.342 for perfluorinated fiber (CYTOP). The fiber diameter is *ρ*_0_ = 62.5, L = 4 mm and *λ* = 650 nm and *n_ex_* = 1.330. In Figure 2 the theoretical absorbance *vs.* the taper ratio for cladded glass fiber, uncladded glass fiber and plastic fiber tapers are reported.

The taper sensitivity increases when tapering increases. Compared to cladded glass taper, the sensitivity of perfluorinated taper (CYTOP) increases faster, especially in the high R region; for example, cladded POF taper with *R* = 0.7 could be about 10 times more sensitive than the cladded glass one. Due to low refractive index of the core/cladding materials perfluorinated tapers also exhibit a higher sensitivity than cladded PMMA tapers. For comparison, the Figure also shows the absorbance of an uncladded glass fiber with the same fiber diameter. As can be observed for taper ratios higher than *R* ≈ 0.36 the sensitivity of perfluorinated POF tapers is greater than that of the uncladded glass one.

## 3. Taper Fabrication and Characterization

The perfluorinated polymer optical fibers used (Chromis Fiberoptics from Thorlabs Inc.) are graded-index plastic optical fibers (GI-POFs) realized by using an amorphous perfluorinated polymer, polyperfluorobutenylvinyl ether (commercially know as CYTOP^®^) [7]. The core is constituted by doped Cytop, with a refractive index of *n_co_* = 1.356, whereas the cladding is Cytop with a refractive index *n_cl_* = 1.342. Two different core/cladding diameters have been used: 62.5/90 μm (GIPOF62) and 120/160 μm (GIPOF120). Moreover, these fibers present a polycarbonate reinforcement overcladding (490 μm diameter) in order to reduce microbending loss in the GI-POF, and to increase the load-bearing capabilities of the fiber [7].

**Figure 3.**
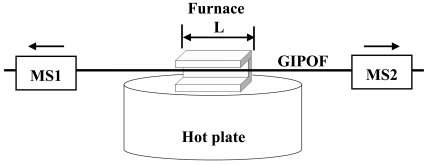
Experimental setup: The GIPOF is heated in the furnace and pulled by two motorized stages (MS1, MS2).

The fabrication process consists of two steps: in the first step, approximately 3.0 cm of the fiber was immersed for several minutes in chloroform for etching the polycarbonate overcladding; then the section without overcladding is heated in a furnace with a length *L* ≈ 1.9 cm and pulled at a fixed velocity using two motorized stages. The furnace is made of a U-shaped aluminum block fixed over a hot plate. This setup, shown in Figure 3, permits a simple insertion and extraction of fiber into the furnace and ensure a controlled extension of the fiber, allowing the prediction of the taper length and the waist dimension [5,12].

**Figure 4.**
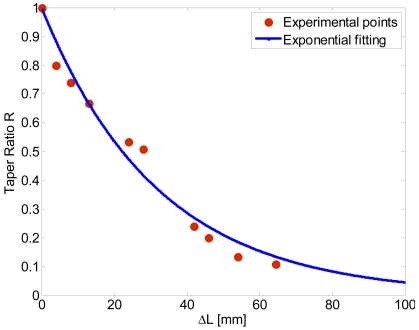
Taper ratio *R* versus the total elongation Δ*L*.

Several experiments have shown that the best results can be obtained with a furnace temperature of about 110 °C (the temperature glass transition of the material is *T_g_* = 108), and setting the stage velocity at 50 μm/s. Devices with different taper ratios have been produced and the repeatability of the process has been verified. In Figure 4 the measured taper ratio *R* versus the total elongation Δ*L* is reported. The taper ratio *R* is predicted to vary with the elongation according to an exponential law [2,5,12]:

(2) R=exp(−ΔL/(2L))

Fitting the measured data with Equation (2) we found a good agreement between theory and experiment for *L* ≈ 1.7 cm (See Figure 4). The small discrepancy with the true furnace length can explained by a non perfect uniform temperature along the furnace due to the open furnace configuration.

Typically the tapers had losses of less than 0.8 dB. This is a good result in comparison with the typical fiber taper losses [5]. We have also characterized the fiber taper shape. If a constant pulling speed is applied, as in our case, the shape of the tapered fiber is independent of the material properties and depends only on the heating profile [5]. In particular, according to theoretical and numerical analysis the taper exhibits a transition region varying exponentially with position over a length Δ*L*/2 and a waist region with an constant diameter at the center with a length *L* [5,12].

The choice of uniform waist tapers allows one to improve the sensitivity compared to parabolic tapers. In fact, the uniform section permits the fabrication of longer tapers and is the region in which the evanescent field has the maximum energy [2].

**Figure 5.**
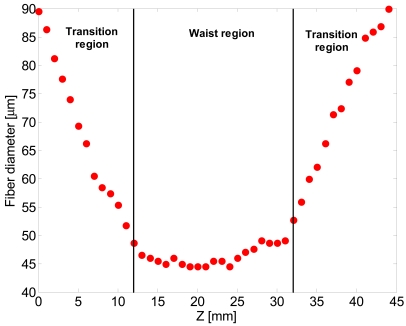
Plot of fiber diameter *vs.* the position on the fiber axis (z axis).

Fiber taper diameter measurements have been carried out by an optical microscope. The experimental result of the tapering process of a GIPOF62 (62.5 μm core and 90 μm cladding) for *R* = 0.5 setting the elongation distance at Δ*L* = 24 mm is shown in Figure 5, where a plot of fiber diameter *vs.* the position on the fiber axis (z axis) is reported. As shown, the taper has an exponential shaped transition, but the in waist region the diameter is not perfectly uniform. These results can be explained with a non-constant temperature along the furnace length as also reported in reference [5].

## 4. Absorbance Measurements

The evanescent absorption properties of both GI-POF120 and GI-POF62 fibers have been investigated. For the GI-POF120 two taper ratios *R* = 0.5 and *R* = 0.75 have been tested. For the GIPOF62, instead, only one taper with taper ratio *R* = 0.5 has been studied.

Absorbance measurements have been performed by immerging the taper region of GIPOF in a plastic sample chamber containing water with variable concentration of methylene blue in the 5 × 10^−8^ M to 1 × 10^−5^ M range.

**Figure 6.**
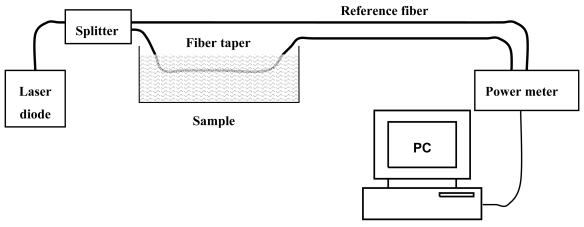
Experimental setup for evanescent wave absorption measurements.

The experimental setup for evanescent wave absorption measurements is reported in Figure 6. Laser light from a fiber-coupled diode laser (*λ* = 650 nm, *P* = 2.5 mW, N.A. = 0.12) has been split using a 1 × 2 fiber splitter (2 m long, 200 μm core diameter, N.A. = 0.22).

**Figure 7.**
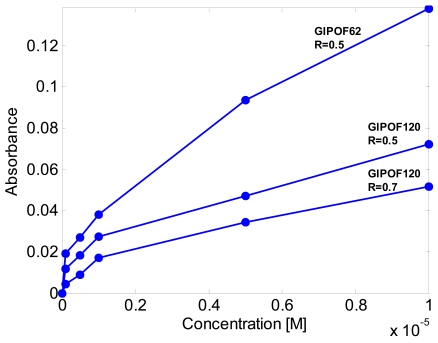
Experimental results of the absorbance measurements

Half of the laser transmission is passed through fiber taper immersed in the absorption test chamber and measured using a photodiode. The other half is measured without transmission through the chamber using a reference photodiode. The signal has been taken as the ratio of the transmitted power from the taper to the reference power. In this way, fluctuations of the optical source have been cancelled. Experimental results for the GIPOF120 with taper ratios *R* = 0.5 and *R* = 0.75 are shown in Figure 7. As can be observed, the absorbance increases as the taper ration decreases.

These results show also that the absorbance is nonlinear with concentration. Assuming a square root dependence of absorbance on concentration, as also reported in other evanescent absorption measurements [13], a least square analysis has been performed with good results, as reported in Table 1.

Finally we have characterized the GIPOF62 with taper ratio *R* = 0.5. From Figure 7 it is clear that for a fixed taper ratio the sensitivity of the taper increases as the core diameter of the fiber ρ_0_ decreases.

**Figure 8.**
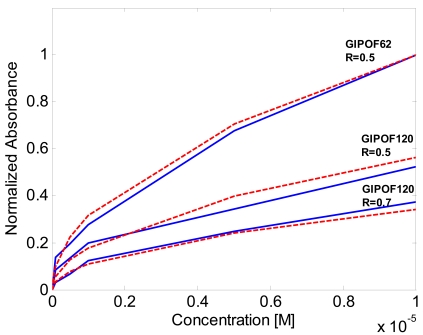
Normalized absorbance verus methylene blue. Experimental results (solid line). Theoretical model (dashed line).

These results have been compared with the ray-optic theory developed by Gou and Albin [3] reported in Section 2. Because the authors, for the calculation, suppose a cladded multimode step-index fiber tapers this approach can provide only qualitative results on the performance of our graded index cladded POF tapers. The results of the simulations are reported in Figure 8 (dashed line). In order to permits a simple comparison between simulations and experimental results we have reported the absorbance normalized to the maximum value. As can be observed there is good qualitative accordance between experimental data and simulations.

## 5. Fluorescence Measurements

Fiber optics tapers have been also proposed for evanescent wave fluoresce collection [9-11]. Typically, uncladded fibers tapers have been used. Here we demonstrate that cladded ones can also be usefully applied. In fact, the fluorescence emitted by a fluorofores located near the fiber coupled into the propagating cladding modes in the taper region. At the end of the taper, some of the cladding modes are coupled back into core modes, hence the collected fluorescence can be detected at the proximal end of the fiber.

**Figure 9.**
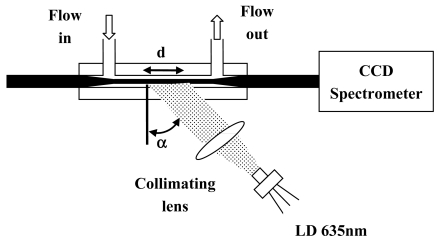
Measurements setup for fluorescence measurements.

Fluorescence measurements have been performed with the experimental setup reported in Figure 9. The sensing features of GI-POF120 fiber taper with a taper ratio *R* = 0.5 has been tested. After the fabrication, the POF taper has been integrated into a microfluidic chamber. Aqueous solutions containing Cy5 dye in a concentration range 3.14 × 10^−7^ M to 6.76 × 10^−6^ M have been used.

The light, from a laser diode, at wavelengths of *λ* = 635 nm has been lunched from the side onto the POF taper. The beam of laser diode has been widened and collimated. The fiber is illuminated at an angle *α* = 60 deg in order to minimize the fluorescence to the background signal due to the scattered excitation light. Only the waist region of the taper (*d* ≈ 2 cm) has been illuminated. The fluorescence light coupled into propagating modes in the taper region has been detected by a CCD spectrometer at the end of the fiber.

**Figure 10.**
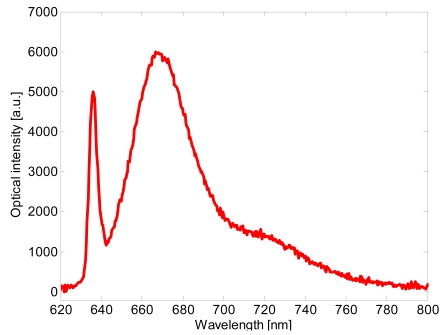
Fluorescence spectrum collected for a Cy5 concentration 6.76 × 10^−6^ M.

Figure 10 shows the collected spectra for a sample concentration of 6.76 × 10^−6^ M. The spectra of the collected fluorescence are given by the optical intensity for *λ* > 650 nm, whereas the narrow peak at 635 nm is the scattered contribution coming from the source. As can be observed, collected scattered excitation light is low compared to the fluorescence.

This is an important result taking into account that an important feature in fluorescence measurements is the ratio between the fluorescent signal and the background signal and the background signal is essentially due to the scattering of the excitation light.

**Figure 11.**
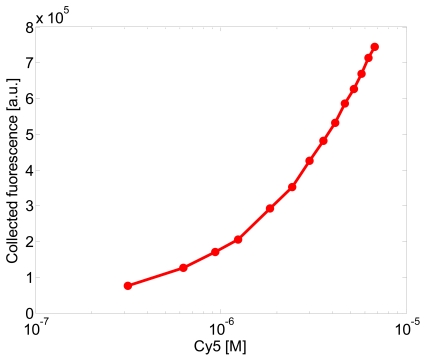
Fluorescence collected for a sample concentration in the 3.14 × 10^−7^ M to 6.76 × 10^−6^ M range.

Figure 11 shows the calibration curves obtained. Each point is the emitted fluorescence evaluated as the sum of the optical intensity detected by the CCD between 650 and 780 nm. These results clearly show that the cladded fiber taper can efficiently collect the fluorescence light also at low concentration.

The limit of detection (LOD) determined using three times the standard deviation at zero concentration is 3.91 × 10^−7^ M.

## 6. Conclusions

A simple and effective procedure for fabrication perfluorinated cladded plastic optical fiber tapers has been developed. The method permits, with a good approximation, the prediction of the final taper ratio and the taper shape. Experimental characterization of the tapers for evanescent wave absorption and fluorescence spectroscopy has been carried out. It has been found that POF cladded tapers permit absorbance and fluorescence measurements with sensitivity comparable or higher to conventional uncladded glass fibers [13-14]. These results show that GIPOF tapers can be used for developing low-cost chemical or biological sensors.

## Figures and Tables

**Table 1.** Least squares regression of absorbance versus square root of concentration.

**Fiber taper**	**Coefficient of correlation**
GIPOF 120 *R* = 0.7	0.997
GIPOF 120 *R* = 0.5	0.997

GIPOF 62 *R* = 0.5	0.994

## References

[1] Mignani A.G., Falciai R., Ciaccheri L. (1998). Evanescent wave absorption spectroscopy by means of bi-tapered multimode optical fibers. Appl. Spectrosc..

[2] Villatoro J., Monzòn-Hernàndez D., Luna-Moreno D. (2004). In-line optical fiber sensors based on cladded multimode tapered fibers. Appl. Opt..

[3] Guo S., Albin S. (2003). Trasmission property and evanescent wave absorption of cladded multimode fiber tapers. Optics Express.

[4] Merchant D.F., Scully P.J., Schmitt N.F. (1999). Chemical tapering of polymer optical fibre. Sens. Actuat. A.

[5] Xue S., van Eijkelenborg M.A., Barton G.W., Hamble P. (2007). Theoretical, numerical, and experimental analysis of optical fiber tapering. J. Lightwave Technol..

[6] Arrue J., Jiménez F., Aldabaldetreku G., Durana G., Zubia J., Lomer M., Mateo J. (2008). Analysis of the use of tapered graded-index polymer optical fibers for refractive-index sensors. Opt. Express.

[7] White W., Blyler L.L., Ratnagiri R., Park M. Manufacture of perfluorinated plastic optical fibers.

[8] Bosch M.E., Sánchez-Ruiz A.J., Rojas F.S., Ojeda C.B. (2007). Recent development in optical fiber biosensors. Sensors.

[9] Taitt C.R., Anderson G.P., Ligler F.S. (2005). Evanescent wave fluorescence biosensor. Biosens. Bioelectron..

[10] Long F., He M., Shi H.C., Zhu A.N. (2008). Development of evanescent wave all-fiber immunosensor for environmental water analysis. Biosens. Bioelectron..

[11] Golden J.P., Anderson G.P., Rabbany S.Y., Ligler F.S. (1994). An evanescent wave biosensor—Part II: Fluorescent signal acquisition from tapered fiber optic probes. IEEE Trans. Biomed. Eng..

[12] Birks T.A., Li Y.W. (1992). The shape of fiber tapers. J. Lightwave Technol..

[13] Ruddy V., MacCraith B.D., Murphy J.A. (1990). Evanescent wave absorption-spectroscopy using multimode fibers. J. App. Phys..

[14] Wu Y., Deng X., Li F., Zhuang X. (2007). Less-mode optic fiber evanescent wave absorbing sensor: Parameter design for high sensitivity liquid detection. Sens. Actuat. B.

